# P-530. Clinician Utilization of CDC-aligned HIV PrEP Panels

**DOI:** 10.1093/ofid/ofae631.729

**Published:** 2025-01-29

**Authors:** Charles M Walworth, Laura Gillim, David Alfego, Ato Aikins

**Affiliations:** Monogram Biosciences/LabCorp, Laguna Beach, California; Labcorp, Burlington, North Carolina; Labcorp, Burlington, North Carolina; Labcorp, Burlington, North Carolina

## Abstract

**Background:**

In Dec 2021, the CDC issued updated guidelines for HIV PrEP. Diagnostic panels were developed to facilitate the baseline evaluation and monitoring of patients for HIV, STIs, HBV, and HCV among PrEP users.

**Methods:**

Retrospective analysis of results reported for HIV PrEP panels ordered between Feb 2023 and Mar 2024. The panels were developed to capture HIV PrEP testing for baseline (BL) evaluation and for monitoring of oral and injectable agents in males and females. Tests were aligned with those recommended by the 2021 updated guidelines and included panels with and without HIV-1 RNA PCR. Standalone testing was also available. Testing intervals were assessed.

**Results:**

31,347 unique patients had PrEP-related testing. Of those, 25,373 had one or more tests with 146 (0.6%) testing positive for HIV at baseline. 14,480 unique patients had two or more HIV PrEP tests on different dates. 98% were male with a median age of 36 years. 20 of those patients tested positive for HIV (0.14%). Of those, 15 tested positive for HIV Ag/Ab, but 14 were negative for HIV RNA. 5 patients were positive for HIV-1 RNA, but tested negative for HIV Ag/Ab. The HIV false positive rate was 0.25%. There were 5 HIV RNA positive tests out of 52,050 negative HIV Ag/Ab tests (0.001%). Testing closely adhered to recommended intervals for both oral and injectable agents. 15,041 out of 25,227 patients (59.6%) had an STI test during the analysis. 2,876 out of 15,041 patients had at least one STI during PrEP treatment (19.1%). Chlamydia was diagnosed in 5.8% and gonorrhea in 9.2%. 6.7% of patients were positive for treponemal antibodies with 5.1% RPR reactive. STI co-infections were also seen. Those with active HBV and HCV infections while on PrEP were each 0.3%.

**Conclusion:**

In this large laboratory cohort of patients utilizing HIV PrEP testing, HIV infections were rare, supporting the low rates seen in HIV PrEP trials. Infections were mainly found at BL screening. False positive HIV Ag/Ab results were consistent with published reports. STI rates are significant with approximately 1/5 of patients testing positive, although active HBV and HCV infections were very low for those utilizing PrEP.

**Disclosures:**

**Charles M. Walworth, MD**, Labcorp: Employee|Labcorp: Employee|Labcorp: Stocks/Bonds (Public Company) **Laura Gillim, PhD**, Labcorp: Employee|Labcorp: Stocks/Bonds (Public Company) **David Alfego, PhD**, Labcorp: Employee|Labcorp: Stocks/Bonds (Public Company) **Ato Aikins, PhD**, Labcorp: Employee

